# Molecular features of androgen-receptor low, estrogen receptor-negative breast cancers in the Carolina breast cancer study

**DOI:** 10.1007/s10549-023-07014-x

**Published:** 2023-07-12

**Authors:** Nikita D. Jinna, Sarah Van Alsten, Padmashree Rida, Victoria L. Seewaldt, Melissa A. Troester

**Affiliations:** 1 Department of Population Sciences, City of Hope Beckman Research Institute, Duarte, CA 91010 USA; 2 Department of Epidemiology, University of North Carolina, Chapel Hill, NC 27599 USA; 3 Department of Science, Rowland Hall, Salt Lake City, UT 84102 USA

**Keywords:** Androgen receptor, Estrogen receptor, Triple-negative breast cancer, Multigene signature, DNA repair, Carolina breast cancer study

## Abstract

**Purpose:**

Androgen receptor (AR) expression is absent in 40–90% of estrogen receptor (ER)-negative breast cancers. The prognostic value of AR in ER-negative patients and therapeutic targets for patients absent in AR remains poorly explored.

**Methods:**

We used an RNA-based multigene classifier to identify AR-low and AR-high ER-negative participants in the Carolina Breast Cancer Study (CBCS; *N* = 669) and The Cancer Genome Atlas (TCGA; *N* = 237). We compared AR-defined subgroups by demographics, tumor characteristics, and established molecular signatures [PAM50 risk of recurrence (ROR), homologous recombination deficiency (HRD), and immune response].

**Results:**

AR-low tumors were more prevalent among younger (RFD =  + 10%, 95% CI = 4% to 16%) participants in CBCS and were associated with HER2 negativity (RFD = − 35%, 95% CI = − 44% to − 26%), higher grade (RFD =  + 17%, 95% CI = 8% to 26%), and higher risk of recurrence scores (RFD =  + 22%, 95% CI = 16.1% to 28%), with similar results in TCGA. The AR-low subgroup was strongly associated with HRD in CBCS (RFD =  + 33.3%, 95% CI = 23.8% to 43.2%) and TCGA (RFD =  + 41.5%, 95% CI = 34.0% to 48.6%). In CBCS, AR-low tumors had high adaptive immune marker expression.

**Conclusion:**

Multigene, RNA-based low AR expression is associated with aggressive disease characteristics as well as DNA repair defects and immune phenotypes, suggesting plausible precision therapies for AR-low, ER-negative patients.

**Supplementary Information:**

The online version contains supplementary material available at 10.1007/s10549-023-07014-x.

## Introduction

Androgen receptor (AR), which is expressed in approximately 30–60% of estrogen receptor (ER)-/progesterone receptor(PR)-/human epidermal growth factor receptor 2(HER2)+ breast cancers and 10–53% of triple-negative breast cancers (ER-/PR-/HER2-; TNBCs) [1–4], has emerged as a candidate therapeutic target for breast cancer patients that lack ER, PR, or HER2 positivity by immunohistochemistry (IHC). Phase 2 clinical trials of AR-targeted therapies such as bicalutamide, abiraterone acetate, and enzalutamide resulted in some clinical benefit [5–7]. However, a large subset (40–90%) of ER-negative patients lack AR expression, and thus are exempt from benefiting from AR-targeted therapies. Furthermore, the prognostic value of AR in ER-negative breast cancer remains uncertain due to conflicting results from multiple studies. Some studies have reported that among TNBCs, lack of AR expression is associated with higher grade, stage, mitotic index, Ki-67, lymph node involvement, younger age at diagnosis, and shorter overall, disease-free, and recurrence-free survival, whereas other groups have reported opposing or no associations with these variables [3, 8–15]. This discordance may be due to differences in the populations studied and to technical factors, including sample procurement, AR antibodies, and cutoffs used for immunohistochemistry, staining protocols, AR production of constitutively active splice variants, and differences in cellular localization [16, 17].

Several research groups have reported that non-luminal androgen receptor TNBC molecular subtypes that express AR protein at low levels demonstrate AR dependence for tumor cell growth or viability [18–21]. Thus, AR IHC may not discern the full range of AR-low states. RNA-based methods allow assessment in large population-based studies with other pathway data. Given the high relative frequency of ER-negative and aggressive tumors in Black women, investigations of AR in diverse populations are needed.

The Carolina breast cancer study (CBCS; phase 1: 1993–1996, 2: 1996–2001; 3: 2008–2013) is a population-based study of breast cancer that is oversampled for Black and younger women. Using gene expression data for 1202 CBCS participants, we trained a pathway-based classifier to identify AR-low patients and to examine the relationship between AR status and tumor aggressiveness among ER-negative participants. Results in CBCS were validated in TCGA.

## Methods

### Study population

The Carolina breast cancer study (CBCS) is a population-based study [22, 23] of women between the ages of 20 and 74 residing in a 24 (CBCS Phases 1 and 2) or 44 (CBCS Phase 3) counties of North Carolina, all of whom were diagnosed with primary invasive breast cancer. Participants were identified through rapid case ascertainment. Black women and women under age 50 were oversampled to achieve a final sample population with approximately 50% Black women and 50% younger women. Race was self-reported. Given that fewer than 2% of the study’s non-Black participants self-identified as a race other than non-Hispanic white, we dichotomized race as Black and non-Black for this study; sensitivity analyses excluding participants self-identifying as something other than Black or white did not change results so we retained these participants to maximize power. Clinical tumor characteristics, including stage, grade, and hormone receptor status (ER and PR), were extracted from medical records and pathology reports. ER and PR status were dichotomized as positive (>1% IHC) or negative (≤1%) in accordance with clinical guidelines, though in sensitivity analyses, we also explored using a 10% cutpoint given that ER-borderline tumors have some features in common with ER negatives [24].

The Cancer Genome Atlas (TCGA) is a large, publicly available data source containing extensive genomic data on over 30 cancer types. Study details are described elsewhere [25]. For our analyses, we downloaded clinical, RNA sequencing, and reverse phase protein array (RPPA) data for 1095 primary breast cancer cases with available RNA data from the NCI Genomic Data Commons (GDC, https://gdc.cancer.gov/). In comparison to cases in CBCS, those in TCGA were older, with larger, higher stage tumors [26].

### Dichotomizing of androgen receptor (AR) RNA expression

1649 CBCS tumor samples, chosen based on the availability of cores or slides for analysis, were included on a NanoString RNA panel that included AR, of which 1202 (72%) passed quality control. Samples which failed quality control were more likely to be from CBCS Phase I or II, while included samples were more likely to be from older participants (M_age_ = 50 vs 52 years), Black women, and ER- tumors (additional details shown in Supplemental Figure 1). Thus, AR RNA expression (alongside that of 416 other RNA-based targets) was profiled in 1202 CBCS samples (472 ER-negatives), using a custom NanoString protocol optimized for formalin-fixed paraffin-embedded (FFPE) samples [27–29]. To accommodate potential study-specific variability in RNA quantification and to address lack of a priori guidelines for categorizing AR expression, cut-points for AR were determined using a mixture model approach among ER-negative breast cancers. Specifically, we estimated 10 Gaussian mixture models, corresponding to the existence of one to nine distinct categories of AR expression, of AR expression values among the 472 ER-negative (absent in ER expression) samples with complete AR data, then selected the final classification model with the lowest BIC [30]. The optimal solution yielded two groups, corresponding to classes with low and high AR expression. We also explored classifications restricted to different indicators of tumor aggressiveness (i.e., age less than 50 years, pre-menopausal status) but found that categorizations restricted to ER-negative samples were a better fit to the data and thus proceeded with an ER/AR-based model (BIC ER = − 1740; BIC Age: − 2474; BIC menopause: − 2330).

**Fig. 1:**
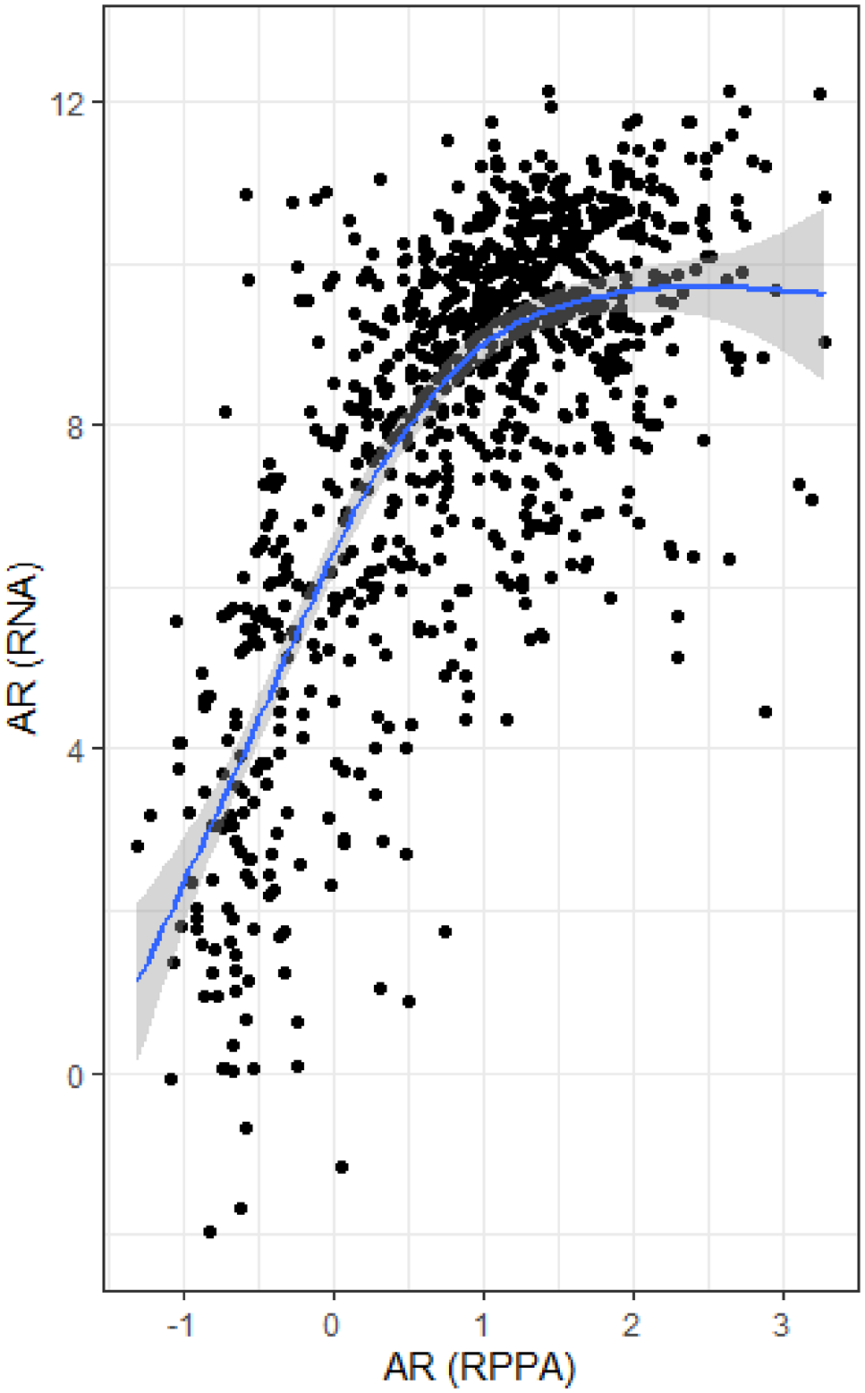
RNA Expression of androgen receptor (AR) correlates with AR protein levels in The Cancer Genome Atlas (*N* = 872)

To identify demographic and clinical features of AR-low tumors, we calculated relative frequency differences (RFDs) and 95% confidence intervals by fitting a generalized linear model with binomial distribution and identity link where AR status was the outcome and the variable of interest was the predictor. Because triple-negative status may confound associations between AR and clinical presentation, we also computed models adjusted for triple-negative status and in triple-negative tumors only. As further sensitivity analyses, we assessed demographic and clinical characteristics of ER+ tumors by AR status to further explore whether AR-low phenotype associations also held for ER+ tumors, and used multiple imputation to assess whether missing data biased results.

### Building a classifier of AR status

To understand overlap between AR-phenotype and deficiencies in immune and DNA repair processes, we analyzed CBCS samples that had RNA expression data on both AR and immune or AR and DNA repair classes. However, because a relatively small number of samples profiled for AR also included information on RNA expression of DNA repair genes (*N* = 674; 271 ER-negative), we developed a predictor of AR phenotype to identify additional samples with low AR expression. To do this, we split the 472 ER-negative samples with measured AR expression into five groups (“folds”) using stratified random sampling, therefore, ensuring consistent distribution of AR-high and AR-low samples within each fold. In each of five iterations of testing, we retained four of the folds for training (training set, *N* = 375–379) and omitted the last for validation (test set, *N* = 93–97), then repeatedly fit Classification to Nearest Centroid (ClaNC) models that used between two and 150 genes (75 models, increasing the number of genes by two each time) to distinguish AR-low and AR-high tumors in the training set. For each of these 75 models, we estimated sensitivity, specificity, and the Youden’s index (sensitivity + specificity − 1) in the training and test sets. We selected the final number of genes to use in the classifier by finding the maximum Youden’s index, averaged across the five folds, among the training sets. From this final model, we predicted the AR status of all ER-negative samples, using predictions to calculate a final sensitivity and specificity. We also assessed model performance by conducting a principal component analysis of RNA expression for the selected genes, coloring samples by AR status to visually inspect how gene expression patterns correlated with AR. Finally, we applied the AR classifier to all ER-negative CBCS samples assayed for selected genes (*N* = 669) and proceeded to compare AR phenotypes to other molecular indicators, described below.

### Associations with molecular signatures

Using custom panels of 50 immune-related and 51 DNA repair-related genes, we classified samples with respect to three immune classes (innate-enriched, adaptive-enriched, and immune-quiet) and two DNA repair classes (recombination/Fanconi anemia (HR/FA), and non-HR/FA) according to published methods [31]). As above, we cross-tabulated AR status with DNA repair status or immune class, and we estimated RFDs and 95% confidence intervals between AR-low and AR-high (referent) samples by fitting a generalized linear model with a binomial distribution and identity link. Positive RFDs indicate enrichment of a given characteristic among AR-low samples. Models were adjusted for TNBC status to determine whether AR status was associated with molecular features independent of TNBC status.

### Validation in TCGA

We used TCGA to validate associations between AR status and molecular tumor characteristics. After applying our ClaNC classifier of AR to ER-negative samples from TCGA, we used the composite Homologous Recombination Deficiency (HRD) Scoring method from Kninjenburg et al. [32] to assess whether AR-low samples were more likely to carry DNA-level evidence of HRD defects than AR-high samples. To confirm associations identified in CBCS, we also compared distributions of DNA repair and immune classes, as defined by the same subsets of genes used in CBCS, across strata of AR status. We also compared AR RNA expression to normalized AR protein levels (*N* = 872 with protein and RNA data) to determine how well findings at the RNA level captured post-translation AR protein status.

## Results

### AR mRNA associations with demographics and clinical features in breast cancer

**Fig. 2:**
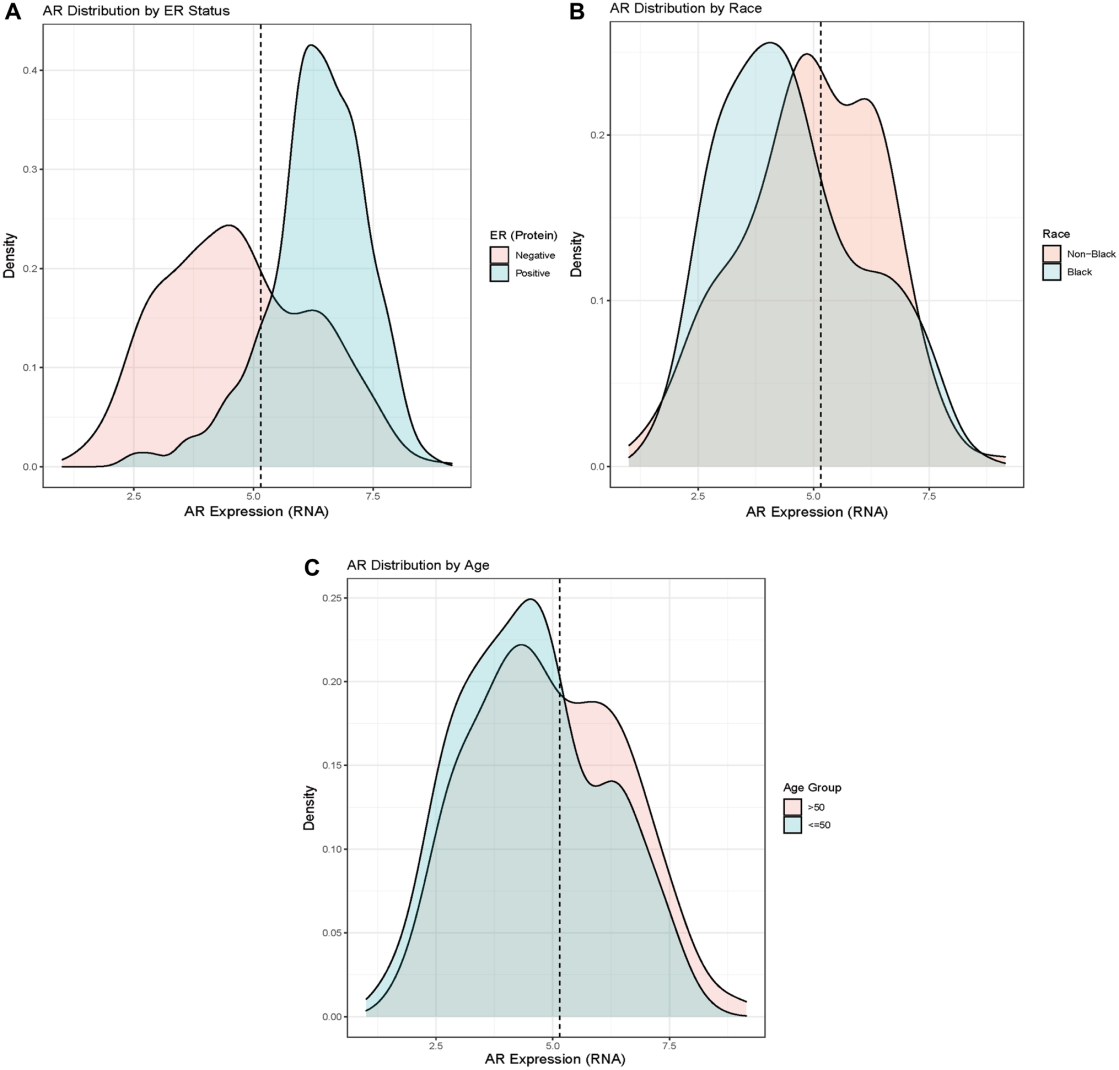
Kernel density estimates of androgen receptor (AR) expression according to estrogen receptor (ER) status in 1202 Carolina breast cancer study participants

We detected associations between single-gene AR RNA expression classes (high vs. low) and aggressive clinical features. RNA levels of AR were strongly correlated with AR protein as measured by RPPA (*r* = 0.68, *p* < 0.001; Fig. 1) in TCGA. Figure 2 shows that lower AR RNA expression was observed in ER-negative tumors (Fig. 2a), and tumors from Black (Fig. 2b) and younger (<50 years old) women (Fig. 2c). Among ER-negative tumors we used model-based density estimation to identify subgroups based on ER expression, which confirmed the presence of two distinct AR expression phenotypes, which we refer to as AR-low/ER- and AR-high/ER-.

ER status assessed via central pathology review of immunohistochemistry. Dashed line is derived from finite mixture model of AR distribution in ER-negative samples and represents cutpoint defining empirical AR groups.

### Clinical and molecular characteristics of ER- breast cancers stratified by AR status

Table 1 shows associations between AR status and selected clinical and molecular characteristics among ER-negative breast cancers. Compared to women with high AR expression (*N* = 168), women with low AR expression (*N* = 304) were more likely to be young, Black, HER2 negative, and high grade. Associations were strongest between AR status and high risk of recurrence genomic scores (ROR-P), with AR-low tumors having a 37% higher prevalence of high ROR-P scores than AR-high tumors (95% CI = 28%–45%). These associations, with the exception of Black race, were somewhat attenuated but remained significant after adjusting for triple-negative status and replicated within triple-negative tumors alone, suggesting that low AR is associated with tumor aggressiveness in ER-negative tumors independent of triple negativity. Similar patterns were observed in ER+ tumors, with AR-low/ER+ tumors being more likely to have high grade, stage, or ROR-P scores than AR-high/ ER+ tumors (Supplementary Table 1). Most ER-borderline tumors were also AR low. Results did not substantially differ after multiple imputation.

**Table 1:** Clinical characteristics of estrogen receptor-negative Carolina breast cancer study participants according to single-gene androgen receptor (AR) RNA expression class

	AR-high (REF)	AR-low	RFD (95% CI)	Adjusted RFD (95% CI)^1^
*N*	168	304		
Age				
> 50 years	70 (41.7)	90 (29.6)	REF	REF
< = 50 years	98 (58.3)	214 (70.4)	12% (3%–22%)	13% (5%–21%)
Race				
White	72 (42.9)	92 (30.3)	REF	REF
Black	96 (57.1)	212 (69.7)	13% (4%–22%)	7% (− 1%–16%)
Menopausal status				
Postmenopausal	89 (53.0)	121 (39.8)	REF	REF
Premenopausal	79 (47.0)	183 (60.2)	12% (4%–21%)	13% (5% – 21%)
Her2				
Negative	104 (63.8)	273 (90.1)	REF	REF
Positive/borderline	59 (36.2)	30 (9.9)	− 38% (− 48%–27%)	− 1% (− 17%–15%)
Grade				
I/II	54 (32.1)	47 (15.5)	REF	REF
III	114 (67.9)	257 (84.5)	23% (12%–33%)	14% (4%–25%)
Stage				
I	38 (23.0)	69 (22.8)	REF	REF
II	86 (52.1)	179 (59.3)	3% (− 7%–14%)	2% (− 8%–12%)
III/IV	41 (24.8)	54 (17.9)	− 8% (− 21%–6%)	− 3% (− 15%–9%)
Tumor size				
< = 2 cm	55 (33.5)	91 (30.2)	REF	REF
> 2–5 cm	77 (47.0)	156 (51.8)	5% (− 5%–15%)	5% (− 5%–14%)
> = 5 cm	32 (19.5)	54 (17.9)	0% (− 13%–13%)	1% (− 11%–12%)
Node status				
Negative	82 (49.7)	180 (59.2)	REF	REF
Positive	83 (50.3)	124 (40.8)	− 9% (− 18%–0%)	− 7% (− 15%–1%)
ROR-P group				
Low/medium	108 (64.3)	79 (26.0)	REF	REF
High	60 (35.7)	225 (74.0)	37% (28%–45%)	27% (18%–36%)

*BMI* body mass index, *ROR*-*P* risk of recurrence proliferation group, *RFD* relative frequency difference, *CI* confidence interval

Clinical characteristics of estrogen receptor-negative Carolina Breast Cancer Study participants according to empirical androgen receptor (AR) status. Relative frequency differences (RFDs) were estimated using generalized linear models with a binomial distribution and identity link function and represent the difference in the proportion of AR-low versus AR-high participants having a given clinical feature. RFD adjusted for triple-negative status

5 participants missing information on stage, 7 missing information on size, 3 on node status

**Fig. 3:**
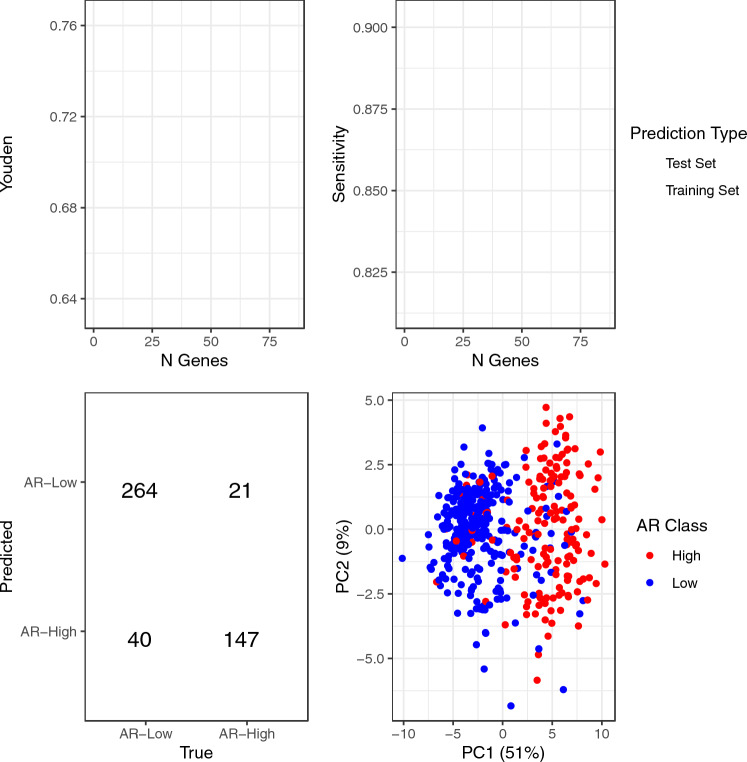
Performance of expression-based low-androgen receptor (AR) classifier in estrogen receptor (ER)-negative samples in the Carolina breast cancer study (CBCS) samples A/B

Given strong associations between low AR and aggressive tumor features, we developed a classifier to identify low AR status using other independent gene expression data when AR expression was missing (Fig. 3). Fivefold cross-validated Classification to Nearest Centroids (ClaNC) showed average sensitivities for identifying AR-low samples among ER-negative tumors in the training set ranging from 82.8% to 88.4%, while specificities ranged from 82.4% to 86.3% (Fig. 3a). The Youden’s index was maximized when using seven genes per AR phenotype (14 genes total), yielding a final sensitivity of 86.8% and specificity of 87.5% (Fig. 3c). Principal components analysis based on the selected genes shows separation of AR low and AR high (Fig. 3d).

Applying the classifier to the 669 ER-negative CBCS tumors with relevant RNA data, we detected 545 (82.8%) tumors with AR-low phenotypes. Again, AR-low tumors were more likely to be younger, Black, HER2-negative, grade III, stages II-IV, and have high ROR-PT scores (Supplementary Table 2) as also observed in the TCGA cohort (Supplementary Table 3). ER/AR-low tumors also showed strong evidence of aggressive molecular phenotypes, with 47.0% having enrichment for adaptive immune tumor subtypes, and 85.6% showing enrichment for homologous recombination-related genes (Table 2). Even after adjusting for triple-negative status, AR-low tumors showed a 33.3% higher frequency of homologous recombination deficient tumors than AR-high tumors, suggesting strong associations between these two tumor phenotypes and that expression of other receptors is not the sole driver of AR-related associations. The magnitude of associations with other (non-adaptive) immune subtypes was less pronounced, although AR-low tumors had 18.5% lower frequency of immune quiet subtype than AR-high tumors.

**Table 2:** DNA repair and immune phenotypes of ER-negative CBCS and TCGA participants according to supervised RNA-based androgen receptor (S-AR) class

CBCS	AR-high (REF)	AR-low	RFD (95% CI)	Adjusted RFD (95% CI)^a^
*N*	124	545		
Immune class				
Adaptive	49 (39.5)	256 (47.0)	REF	REF
Innate	47 (37.9)	240 (44.0)	− 0.3% (− 6.3%–5.6%)	− 0.2% (− 5.0%–4.5%)
Quiet	28 (22.6)	49 (9.0)	− 20.3% (− 32.1%–9.2%)	− 18.5% (− 30%–8.1%)
DNA repair class				
Not HR/FA	54 (62.1)	55 (14.4)	REF	REF
HR/FA	33 (37.9)	326 (85.6)	40.3% (30.5%–50.1%)	33.3% (23.8%–43.2%)

TCGA				
*N*	50	187		
Immune class				
Immune-high	256 (41.2)	225 (47.6)	REF	REF
Immune-low	365 (58.8)	248 (52.4)	− 5.2% (− 15.7%–5.2%)	− 5.5% (− 15.8%–4.9%)
DNA repair class				
Not HR/FA	31 (62.0)	56 (29.9)	REF	REF
HR/FA	19 (38.0)	131 (70.1)	49.2% (43.1%–54.8%)	41.5% (34.0%–48.6%)

Clinical and molecular phenotypes of Carolina Breast Cancer Study participants according to expression-based androgen receptor (AR) classifier status. Relative frequency differences (RFDs) were estimated using generalized linear models with a binomial distribution and identity link function and represent the difference in the proportion of AR-low-like versus AR-high-like participants having a given clinical feature

^a^Adjusted for triple negative status

Applying the classifier to data from ER-negative tumors in TCGA, we identified 187 tumors (58.7% ER +) with AR-low phenotypes. AR-low tumors showed higher frequency of homologous recombination deficiency (HRD), with 81 (39.3%) having HRD scores above the clinical cutoff of 42 as compared to 11 (5.3%) of AR-high tumors. We did not find strong evidence of an association between AR and immune expression phenotypes in TCGA (RFD = − 5.5%, 95% CI = − 15.8%–4.9%), although the TCGA lacks evidence of the immune quiet phenotype due to different selection factors for inclusion in TCGA [26]. However, AR-low tumors again had higher proportions of expression-based HRD phenotypes than AR-high tumors (adjusted RFD: 41.5%, 95% CI = 34.0%–48.6%).

Fivefold cross-validated Youden’s index and sensitivity of Classification to Nearest Centroids classifier according to number of genes used to predict each AR phenotype. Confidence intervals represent mean plus or minus standard error. Blue lines represent predictions in the training set (*N* = 375–389), and red lines represent predictions in the test set (*N* = 93–97). C. Final performance of Youden’s Index-maximizing classifier using seven genes per group. Correct classifications are shown in green, while incorrect classifications are shown in red. Sensitivity was 86.8%, specificity was 87.5%, and overall accuracy was 87.1%. D. Principal component analysis based on RNA expression of classifier-selected genes in ER-negative CBCS samples. Red samples are those with low AR expression, and blue samples have high AR expression.

## Discussion

Limitations and inconsistencies with protein-based AR assessment approaches have been previously cited as a barrier to AR expression interpretation and its consistency in the literature [3, 9–12, 14, 16, 17]. Using CBCS expression data, we designed and validated a multigene classifier that distinguishes AR-low versus AR-high ER-negative breast cancers. AR-low status in ER-negative breast cancer was significantly associated with younger age at diagnosis, Black race, HER2 negativity, high-grade, and higher ROR; these associations, with the exception of Black race, remained significant after adjusting for TNBC status. These findings suggest that in ER-negative breast cancers, low AR expression is associated with aggressive disease. Considering other biological phenotypes, AR-low cancers in the CBCS cohort exhibited adaptive immunity enrichment and both CBCS and TCGA datasets displayed significantly greater homologous recombination repair deficiency among AR-low cancers.

Our findings are consistent with what has been previously reported for demographic factors. Park *et al.* reported that women, under the age of 35 years, were diagnosed with AR-negative/ER-negative breast cancer more frequently than women over the age of 35 (11.7% and 7.0%, respectively) [33]. Several groups have reported that in TNBC, AR-negativity is significantly associated with younger age at diagnosis and that older age at diagnosis is more prevalent in the luminal androgen receptor subtype and among AR-positive TNBC patients [15, 18, 19, 34–36]. Prior evidence also showed that low AR expression in ER-negative cancer is associated with Black race and West African genetic ancestry [37–39].

Our findings also corroborate prior evidence suggesting that AR-negative TNBC is associated with aggressive disease features such as advanced stage and high histological grade [3, 8, 10, 11]. The multi-parametric gene expression-based signature ROR-PT is derived from the PAM50 intrinsic subtype signature and has been reported to predict distant recurrence in node-negative and node-positive ER-positive breast cancer patients [40–44]. Our ROR-PT analysis aligns with previous studies showing higher incidence of recurrence or relapse in women with AR-low versus AR-high ER-negative breast cancer [8, 12, 45–48]. Yang et al. reported that AR positivity is associated with longer relapse-free survival among HER2-negative patients [49]. Wang and colleagues showed that women with AR-low TNBC, a subset of AR-low ER-negative breast cancer, exhibited a greater incidence of distant metastases than women with AR-high TNBC [50].

Another distinction observed herein between AR-negative and AR-positive TNBC was with respect to immune profiles. Consistent with our findings, Davis and colleagues previously reported that AR-negative tumors are upregulated in T-cell marker (CD4 and CD8), immune checkpoint (PD1, PD-L1, and CTLA-4), and immune cell-signaling pathway marker (ILR2, CCR5, NFKBII2) RNA expression compared to AR-positive tumors in TNBC [51]. These findings suggest that AR-negative TNBC may display increased numbers of infiltrating lymphocytes, but exhibit greater immunosuppression compared to AR-positive TNBC. Our RNA-based classifier identified enrichment in adaptive immunity in AR-low compared to AR-high ER-negative tumors.

Previous studies have not evaluated associations between AR and specific DNA repair pathways. We observed a higher prevalence of homologous recombination deficiency in AR-low (versus AR-positive) ER-negative-breast cancer. This finding is consistent with previous studies showing increased genomic instability in AR-negative (versus AR-positive) TNBC. It has been shown that AR-negative TNBCs have 1) increased epidermal growth factor receptor, cyclin-dependent kinase 6, Ki-67, and topoisomerase 2a but 2) downregulated PTEN and HER4 [52–55]. It was also discovered that AR-negative tumors display a higher level of copy number alterations (CNAs), CIN25, centrosome amplification, and miRNAs/mRNAs pairings associated with genomic instability, cell cycle, and DNA damage [56]. Thus, AR negativity in TNBC may be linked to dysregulation in the cell cycle and impaired DNA damage response, specifically homologous recombination.

Due to study-specific differences in normalization methods and RNA sequencing protocols, the cutoffs used in our analysis may not translate directly to other analyses, though RNA-based classifiers and mixture models should yield similar results. Another limitation was the small sample size of ER-negative breast cancer patient participants, particularly and TNBC patients. QNBC is a subgroup of TNBC that lacks AR expression and has been reported to be more biologically aggressive and distinct from AR-positive TNBCs [51]. A larger pool of QNBC participants would improve our precision and enable analysis of associations with survival and recurrence among AR-low and AR-high subgroups of TNBC. We were also unable to compare directly to IHC, but previous studies have shown that AR RNA-based signatures expression do not always correlate with AR protein.

Our study’s findings suggest RNA-based methods may be valuable for identifying AR-low, ER-negative breast cancer patients. This gene expression-based approach may capture AR-dependent tumor biology that has been inconsistently observed based on IHC alone. Other research groups have designed multigene signatures that predict response to AR-therapy better than biomarker-based IHC alone [57, 58]. In a 2015 phase II study, Traina and colleagues showed that a novel binary multigene biomarker was a better predictor of enzalutamide response than AR expression by IHC [58]. In 2019, Nyquist et al. showed that a multigene signature indicated response to AR-targeted therapies better than monogenic biomarkers [57]. Thus, results from our group and others indicate that RNA-based signatures may have value for capturing AR levels in ER-negative patients. Our findings also suggest that AR-low ER-negative patients may be more likely to have biological features that have previously indicated response to DNA damage-based agents and immunotherapy. Future research may investigate AR as a prognostic biomarker for chemotherapy- or immunotherapy-treated breast cancer patients.”

## Supplementary Information

Below is the link to the electronic supplementary material.

Supplementary file1 (DOCX 24 KB)

Supplementary file2 (PDF 79 KB)

## Data Availability

The datasets generated during the current study are available from the corresponding author upon reasonable request. The code in this study is available from the corresponding author upon reasonable request.
