# Supplementary material for: Molecular features of androgen-receptor low, estrogen receptor-negative breast cancers in the Carolina breast cancer study
Source: Breast Cancer Res Treat. 2023 Jul 12;201(2):171–81. doi: 10.1007/s10549-023-07014-x (PMC10361868; doi:10.1007/s10549-023-07014-x)
Supplement: Supplementary file 1 — Supplementary file1 (DOCX 24 KB) [file 10549_2023_7014_MOESM1_ESM.docx]

**Supplementary Table 1.** Distribution of demographic and clinical variables by cross-classified ER and AR status in the Carolina Breast Cancer Study.

|  | ER-/AR- | ER-/AR+ | ER+/AR- | ER+/AR+ | p |
| --- | --- | --- | --- | --- | --- |
|  | 304 | 168 | 96 | 626 |  |
| Stage |  |  |  |  |  |
| I | 69 (22.8) | 38 (23.0) | 27 (28.4) | 240 (39.0) | <0.001 |
| II | 179 (59.3) | 86 (52.1) | 50 (52.6) | 284 (46.1) |  |
| III/IV | 54 (17.9) | 41 (24.8) | 18 (18.9) | 92 (14.9) |  |
| Grade |  |  |  |  |  |
| I/II | 47 (15.5) | 54 (32.1) | 53 (55.2) | 473 (75.6) | <0.001 |
| III | 257 (84.5) | 114 (67.9) | 43 (44.8) | 153 (24.4) |  |
| Node Status |  |  |  |  |  |
| Negative | 180 (59.2) | 82 (49.7) | 54 (56.2) | 342 (54.7) | 0.251 |
| Positive | 124 (40.8) | 83 (50.3) | 42 (43.8) | 283 (45.3) |  |
| Tumor Size |  |  |  |  |  |
| <=2 | 91 (30.2) | 55 (33.5) | 35 (36.8) | 314 (51.0) | <0.001 |
| >2-5 | 156 (51.8) | 77 (47.0) | 47 (49.5) | 241 (39.1) |  |
| >5 | 54 (17.9) | 32 (19.5) | 13 (13.7) | 61 ( 9.9) |  |
| ROR-P Group |  |  |  |  |  |
| Low/med | 79 (26.0) | 108 (64.3) | 47 (49.0) | 573 (91.5) | <0.001 |
| High | 225 (74.0) | 60 (35.7) | 49 (51.0) | 53 ( 8.5) |  |

**Supplementary Table 2.** Clinical characteristics of estrogen receptor negative Carolina Breast Cancer Study participants according to classifier-inferred androgen receptor AR) status.

|  | AR-high (REF) | AR-low | RFD (95% CI) | Adjusted RFD (95% CI)^1^ |
| --- | --- | --- | --- | --- |
| N | 124 | 545 |  |  |
| Age |  |  |  |  |
| > 50 years | 62 (50.0) | 189 (34.7) | REF | REF |
| <= 50 years | 62 (50.0) | 356 (65.3) | 10% (4% - 16%) | 11% (6% - 17%) |
| Race |  |  |  |  |
| White | 52 (41.9) | 173 (31.7) | REF | REF |
| Black | 72 (58.1) | 372 (68.3) | 7% (1% - 14%) | 3% (-3% – 8%) |
| Menopausal Status |  |  |  |  |
| Postmenopausal | 68 (54.8) | 247 (45.3) | REF | REF |
| Premenopausal | 56 (45.2) | 298 (54.7) | 6% (0% - 12%) | 8% (3% -13%) |
| Her2 |  |  |  |  |
| Negative | 62 (50.8) | 476 (87.3) | REF | REF |
| Positive/Borderline | 61 (49.2) | 69 (12.7) | -35% (-44% - -26%) | -16% (-30% - -1%) |
| Grade |  |  |  |  |
| I/II | 41 (33.1) | 87 (16.0) | REF | REF |
| III | 83 (66.9) | 458 (84.0) | 17% (8% - 26%) | 13% (6% - 21%) |
| Stage |  |  |  |  |
| I | 31 (25.2) | 133 (24.6) | REF | REF |
| II | 56 (45.5) | 308 (56.9) | 4% (-3% – 11%) | 3% (-2% - 9%) |
| III/IV | 36 (29.3) | 100 (18.5) | -8% (-17% - 2%) | -5% (-13% - 4%) |
| Tumor Size |  |  |  |  |
| <=2cm | 49 (39.8) | 181 (33.6) | REF | REF |
| >2 - 5cm | 54 (43.9) | 272 (50.5) | 5% (-2% - 12%) | 5% (-1% - 10%) |
| >=5cm | 20 (16.3) | 86 (16.0) | 2% (-7% - 11%) | 1% (-8% - 8%) |
| Node Status |  |  |  |  |
| Negative | 55 (44.4) | 324 (59.9) | REF | REF |
| Positive | 69 (55.6) | 217 (40.1) | -10% (-16% - 4%) | -7% (-12% - -1%) |
| ROR-P Group |  |  |  |  |
| Low/Medium | 85 (70.8) | 188 (34.6) | REF | REF |
| High | 35 (29.2) | 355 (65.4) | 22% (16.1% - 28%) | 17% (11% - 23%) |

Note: 5 participants missing information on stage, 7 missing information on size, 4 on node status, and 6 on ROR-P group.

Abbreviations: ROR-P- risk of recurrence proliferation group; RFD- relative frequency difference; CI- confidence interval.

1. RFD adjusted for triple negative status.

**Supplementary Table 3.** Clinical characteristics of TCGA participants according to classifier-inferred androgen receptor (AR) status.

|  | AR-high (REF) | AR-low | P value |
| --- | --- | --- | --- |
| N | 621 | 473 |  |
| ER Status |  |  | <0.001 |
| Positive | 543 (91.6) | 264 (58.5) |  |
| Negative | 50 ( 8.4) | 187 (41.5) |  |
| Age |  |  | <0.001 |
| > 50 years | 466 (75.2) | 298 (63.0) |  |
| <= 50 years | 154 (24.8) | 175 (37.0) |  |
| Race |  |  | 0.005 |
| White | 447 (83.7) | 307 (76.2) |  |
| Black | 87 (16.3) | 96 (23.8) |  |
| Menopausal Status |  |  | 0.018 |
| Postmenopausal | 422 (78.6) | 283 (71.6) |  |
| Premenopausal | 115 (21.4) | 112 (28.4) |  |
| Stage |  |  | 0.95 |
| I | 104 (17.2) | 78 (16.8) |  |
| II | 348 (57.4) | 271 (58.4) |  |
| III/IV | 154 (25.4) | 115 (24.8) |  |
| Node Status |  |  | 0.096 |
| Negative | 244 (46.7) | 212 (52.5) |  |
| Positive | 278 (53.3) | 192 (47.5) |  |
| ROR-P Group |  |  | <0.001 |
| Low/Medium | 564 (90.8) | 303 (64.1) |  |
| High | 57 ( 9.2) | 170 (35.9) |  |
|  |  |  |  |
|  |  |  |  |
