# Supplementary figures and images for: Molecular features of androgen-receptor low, estrogen receptor-negative breast cancers in the Carolina breast cancer study
Source: Breast Cancer Res Treat. 2023 Jul 12;201(2):171–81. doi: 10.1007/s10549-023-07014-x (PMC10361868; doi:10.1007/s10549-023-07014-x)

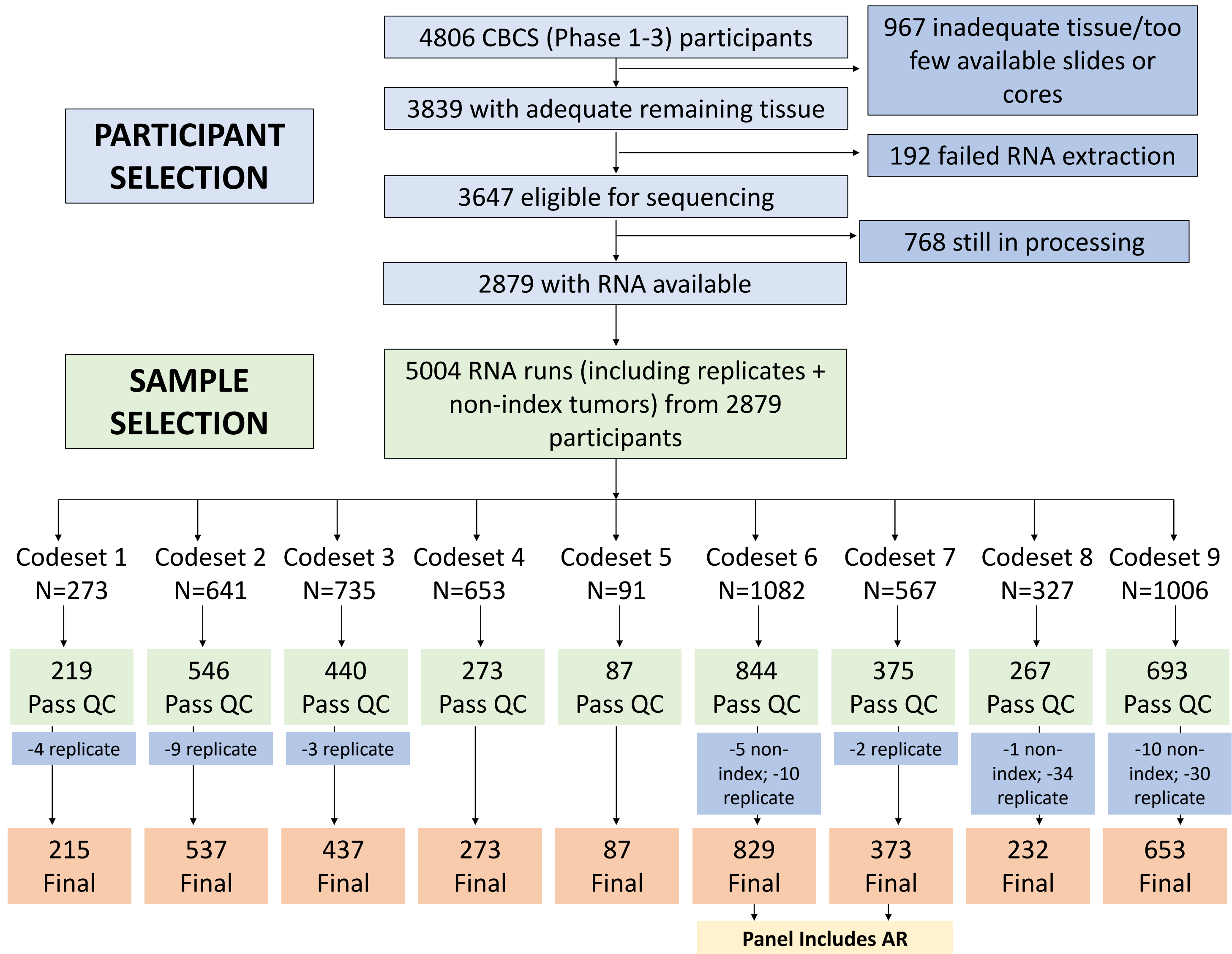

Supplement: Supplementary file 2 — Supplementary file2 (PDF 79 KB) [file 10549_2023_7014_MOESM2_ESM.pdf]
